# NDRG4 is a novel oncogenic protein and p53 associated regulator of apoptosis in malignant meningioma cells

**DOI:** 10.18632/oncotarget.4009

**Published:** 2015-05-25

**Authors:** Rama P. Kotipatruni, Xuan Ren, Dinesh Thotala, Jerry J. Jaboin

**Affiliations:** ^1^ Department of Radiation Oncology, Cancer Biology Division, School of Medicine, Washington University in Saint Louis, St. Louis, Missouri, USA; ^2^ Siteman Cancer Center, Washington University in Saint Louis, St. Louis, Missouri, USA

**Keywords:** meningioma, NDRG4, p53, lentiviral shRNA, mitochondrial apoptosis

## Abstract

Aggressive meningiomas exhibit high levels of recurrence, morbidity and mortality. When surgical and radiation options are exhausted, there is need for novel molecularly-targeted therapies. We have recently identified NDRG4 overexpression in aggressive meningiomas. NDRG4 is a member of the N-Myc Downstream Regulated Gene (NDRG) family of the alpha/beta hydrolase superfamily. We have demonstrated that NDRG4 downregulation results in decreased cell proliferation, migration and invasion. In follow up to our prior studies; here we demonstrate that the predominant form of cell death following NDRG4 silencing is apoptosis, utilizing Annexin-V flow cytometry assay. We show that apoptosis caused by p53 upregulation, phosphorylation at Ser15, BAX activation, Bcl-2 and BcL-xL downregulation, mitochondrial cytochrome c release and execution of caspases following NDRG4 depletion. Sub-cellular distribution of BAX and cytochrome c indicated mitochondrial-mediated apoptosis. In addition, we carried out the fluorescence cytochemical analysis to confirm mitochondrial-mediated apoptosis by changes in mitochondrial membrane potential (Ψm), using JC-1 dye. Immunoprecipitation and immunofluorescence confirmed binding of NDRG4 to p53. In addition, we demonstrate that apoptosis is mitochondrial and p53 dependent. The proapoptotic effect of p53 was verified by the results in which a small molecule compound PFT-α, an inhibitor of p53 phosphorylation, is greatly protected against targeting NDRG4 induced apoptosis. These findings bring novel insight to the roles of NDRG4 in meningioma progression. A better understanding of this pathway and its role in meningioma carcinogenesis and cell biology is promising for the development of novel therapeutic targets for the management of aggressive meningiomas.

## INTRODUCTION

Meningiomas represent one third of all primary central nervous system neoplasms [1]. They are the second most common brain tumors, and originate from the arachnoid “cap” cells of the arachnoid villi [2, 3]. While most meningiomas are benign, up to 15% are aggressive and characterized by a capacity for normal brain invasion with frequent and destructive recurrence patterns. Maximally safe surgical resection is the standard of care for all subtypes and grades of meningioma with adjuvant radiotherapy often used for aggressive tumor variants, or recurrent WHO Grade I tumors [4, 5]. WHO Grade II has a recurrence rate of greater than 40%, and WHO Grade III tumors recur over 80% of the time with death occurring within a decade after diagnosis [6, 7]. Malignant tumor progression depends upon the capacity of tumor cells to invade, metastasize and promote an angiogenic host response. Though surgery and radiation are important to therapy, there are few adjuvant therapies available to patients which are unresectable or recur. However, tumor growth rate following radiation therapy remains an intimidating clinical challenge with few effective alternative therapeutic options [8]. Alternative treatment options are required for patients with either malignant or recurrent benign meningiomas that are surgically inaccessible. There is a need for novel molecular targets to improve survival and reduce morbidity for this group of patients. We recently showed that N-Myc down regulated gene 4 (NDRG4) was overexpressed in aggressive meningioma [9]. NDRG4 is involved in modulating cell proliferation, invasion, migration and angiogenesis in meningioma, and may play a valuable role as a molecular target in its treatment.

The N-Myc downstream-regulated gene (NDRG) family consists of NDRG1–4, these are identified as a novel class of Myc repressed intracellular proteins consisting of 340–394 amino acid residues with 57–65% sequence homology [10, 11]. The NDRG family proteins are members of the *alpha/beta* hydrolase super family; these α/β-hydrolases exhibit multiple surface hydrophobic residues that facilitate their molecular interactions [12]. Although the functional role in cellular progression has not yet been identified, NDRG4 have been identified as a novel interaction partner for Bves (Blood vessel epicardial substance). However, these protein-protein interactions have been mostly characterized in epithelial cells that influence epicardial cell movement [13]. NDRG proteins have also been implicated in development [11, 14], cancer metastasis [15, 16], and the immune system [17, 18]. Each of the four NDRG proteins demonstrates a distinct spatiotemporal expression pattern during embryonic development and in adult tissues [19, 20]. NDRG2 and NDRG4 are highly expressed in brain and heart [21] and promote neurite extension in PC12 neuronal cells [22, 23, 24]. Recent literature suggests that NDRG2 interacts with p53 and regulates apoptosis in oxygen-glucose deprived C6-originated astrocytes [25]. This p53 interaction seems to be preserved in human lung, breast and brain malignancies [26].

NDRG4 has roles in development; including zebrafish myocyte proliferation [10] and normal brain development and function [27]. However NDRG4 also been identified as a tumor suppressor gene with NDRG4 overexpression resulting in decreased colorectal cancer cell proliferation and invasion [28]. Most recently, NDRG4 has been found to be upregulated in glioblastoma, suggesting roles in cell cycle regulation and survival [29]. The relationship between NDRG4 and cell survival in meningioma is not established yet *in vivo* but knockdown of NDRG4 decreases migration, invasion and inhibited cell cycle progression in meningioma cells [9].

Cell proliferation and apoptotic cell death are very complex processes that involve the participation of a host of genes. In both events, p53 is one of the most important and studied tumor suppressor genes [30]. P53 maintains tumor suppression by transcriptional regulation of genes involved in cell growth and apoptosis [31]. Elevated levels of p53 are observed in malignant meningiomas and overexpression of p53 is associated with high levels of cellular proliferation, rapid tumor recurrence and radioresistance [32]. The p53 tumor suppressor protein mediates a range of mitochondria mediated apoptotic responses initiated by various external and internal stimuli [33]. The fundamental consequences of mitochondrial-mediated apoptosis include the unstablised mitochondrial membrane integrity, cytochrome c release and the activation of Bcl-2 family proteins [34]. BAX, a pro apoptotic protein is mainly localized in the cytoplasm and translates into mitochondria in response to apoptotic stimuli [35]. The extrinsic pathway of apoptosis requires the cytochrome *c* release from the mitochondrial intermembrane space to the cytosol [36]. Once released, cytochrome *c* cooperates with the adaptor protein, APAF-1, to promote the activation of caspases, which are required for the rapid recognition, triggers DNA fragmentation and clearance of the abnormal cells [37].

Our research involves the discovery of targets that would enhance the effects of meningioma cancer treatment. RNA interference-based, targeted silencing of gene expression is a strategy of potential interest for cancer therapy [38]. Currently, attempts are being made to overcome the adverse effects and limitations of radiation-resistant tumor cells using the gene therapy [39]. However, the detailed contribution of the NDRG4 protein and its biological significance in malignant meningiomas has not been studied. The mechanism of action of the depleted NDRG4 induced cell death was unknown. In the present study, to better characterize the key roles of NDRG4 *in vitro*, we have used a lentiviral vectors expressing small hairpin RNA directed against NDRG4. We demonstrate for the first time that downregulation of NDRG4 gene activates p53 by phoshorylation at Ser^15^, increased BAX expression and translocation into mitochondria, which leads to destabilization of mitochondria which has ultimately induces the activated caspase mediated apoptosis in meningioma tumor cells. It is clear that the understanding of NDRG4 function in the development of meningioma pathology may give new insights in future therapeutic strategies including gene therapy. Hence, selectively targeting NDRG4 with lentiviral mediated gene silencing may be a potential cancer therapy for malignant meningiomas.

## RESULTS

### Knockdown of NDRG4 regulates cell proliferation and induces the apoptosis in meningioma

**Figure 1:**
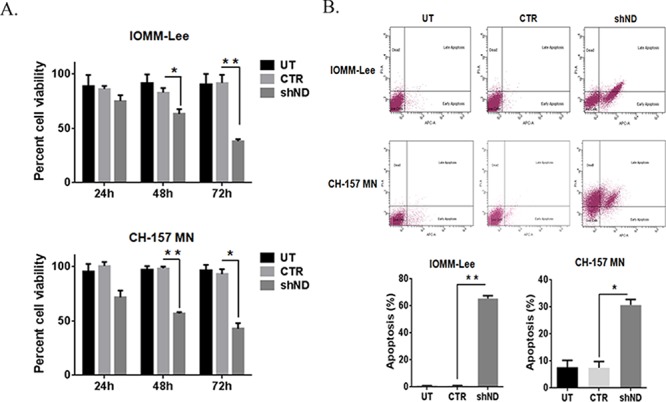
Knockdown of NDRG4 decreases the cell viability and induces the apoptosis **A.** IOMM-Lee and CH-157 MN cells were infected with viruses generated in HEK 293T cells transfected with Control vector (CTR) or NDRG4 (shND) specific shRNA. Equal numbers of IOMM-Lee and CH-157 MN cells were plated in 96-well plates after gene silencing. After 24 hrs, 48 hrs and 72 hrs, cells were treated with MTT and the cell viability was determined using a colorimetric cell proliferation assay. Shown is the percent viability. Error bars indicate SEM (*p < 0.05, *n* = 3). **B.** Suppression of NDRG4 gene expression induces apoptosis in IOMM-Lee and CH-157 MN cells. IOMM-Lee and CH-157 MN cells were infected with lentivirus generated in HEK 293T cells transfected with Control vector or NDRG4 specific shRNA. 72 hrs of post transduction, untreated (UT), control (CTR) and shNDRG4 (shND) treated cells were stained with Annexin V-APC/propidium iodide and analyzed by flow cytometry. The graph indicating the percent apoptosis for each treatment group is shown SEM of triplicates. *p < 0.05.

To evaluate the role of NDRG4 in cell proliferation and cell death in meningioma cells, NDRG4 was targeted in IOMM-Lee and CH-157 MN cells using specific lentiviral shRNA vectors. IOMM-Lee and CHMN-157 are the most frequently used and well characterized high-grade meningioma cell lines. Knockdown of NDRG4 gene was confirmed using immunoblots after viral purification. Targeting of NDRG4 significantly decreased proliferation rates in both IOMM-Lee (74.93%, *p* = 0.1072) at 24 hrs, 63.0%, *p* = 0.0048 at 48 hrs and 38.15%, *p =* 0.0002 at 72 hrs) and CH-157 MN (71.51%, *p =* 0.0108 at 24 hrs, 56.65%, *p =* 0.0001 at 48 hrs and 42.65%, *p* = 0.0002 at 72 hrs) compared to control cells. In both the cell lines targeting NDRG4 significantly decreased proliferation (Figure 1A). We further analyzed these cells for apoptosis using Annexin V-APC assay. Knockdown of NDRG4 induced 43.2% of early apoptosis and 18.4% of late apoptosis in the IOMM-Lee cells and 4% early apoptosis and 29.4% late apoptosis in the CH MN-157 cells was detected. Targeting NDRG4 significantly induced apoptosis in both IOMM-Lee (65%; *p* = 0.0001) and CH-157 MN cells (31%; *p* = 0.0061) when compared to control cells (Figure 1B). These results indicate that NDRG4 regulated proliferation and prevented apoptosis in IOMM-Lee and CH-157 MN cells.

### NDRG4 regulates apoptotic signaling in meningioma cells

**Figure 2:**
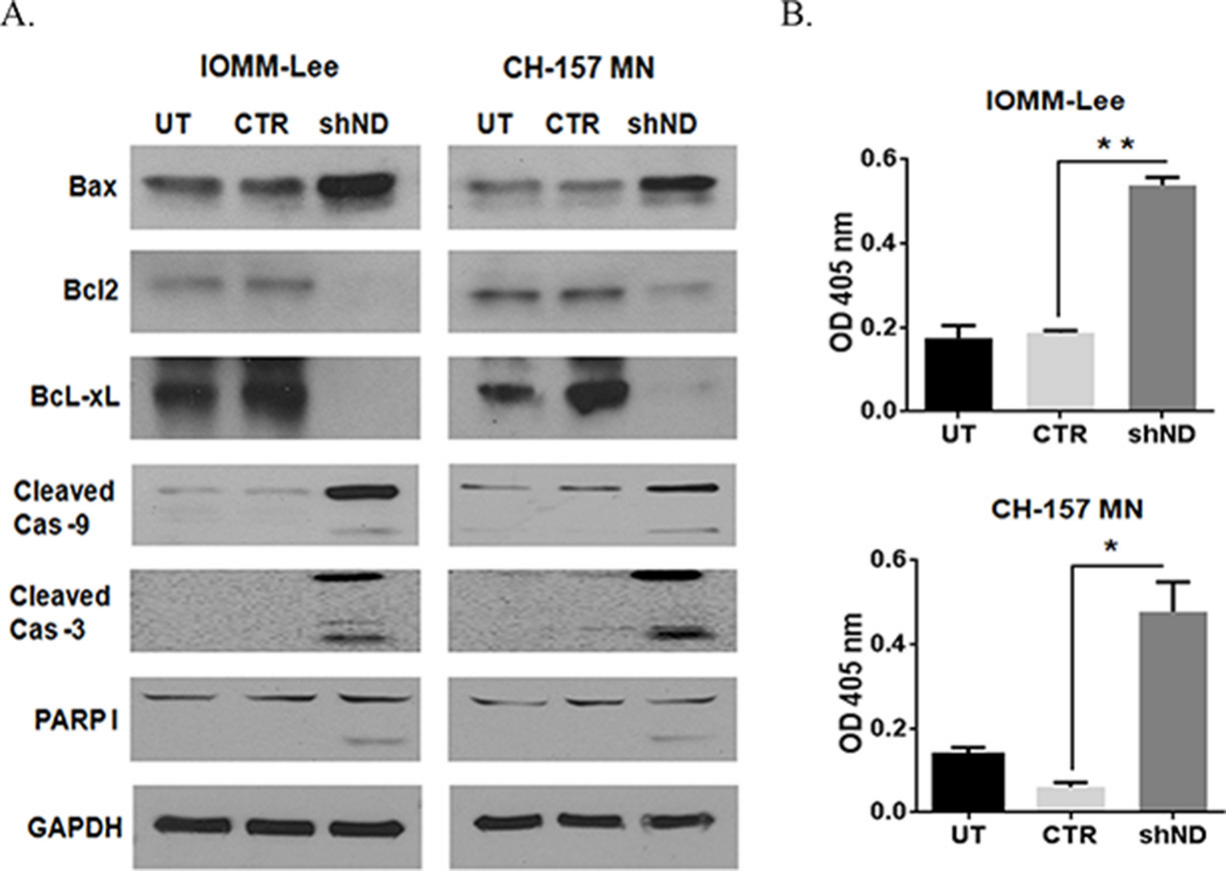
Silencing NDRG4 induces apoptosis through the mitochondrial apoptotic pathway **A.** IOMM-Lee and CH-157 MN cells were infected with lentivirus generated in HEK 293T cells transfected with control vector or NDRG4 specific shRNA. The knockdowned cells were lysed using RIPA/MPER buffer. 40 μg cell lysate of mitochondrial apoptotic proteins: BAX, BcL2, BcL-xL, cleaved Caspase 9, cleaved Caspase 3 and PARP I were immunoblotted. **B.** Caspase 9 enzyme activity assay. IOMM lee and CH-157 MN cells were infected with viruses generated in HEK 293T cells transfected with control vector or NDRG4 specific shRNA. 72 hrs of post transduction, untreated, control and shNDRG4 treated cells were lysed using RIPA/MPER and 40 μg of cell lysate were incubated with a LEHD-*p*NA substrate. Caspase 9 activity was measured at 405 nm. The graph indicating the OD at 405 nm for each treatment group is shown in triplicates. Error bars indicate SEM (*p < 0.05, *n* = 3).

To determine the role of NDRG4 in the molecular mechanism of apoptosis, we analyzed mitochondrial Bcl-2 family protein expression in presence or absence of NDRG4 in meningioma cell lines by western Immunoblotting, 72 hrs post infection. Targeting NDRG4 in IOMM-Lee cells and CH-157 MN cells led to increased accumulation of pro apoptotic protein BAX and decrease in anti-apoptotic proteins Bcl-2 and Bcl-xL. Targeting of NDRG4 also led to increased caspase 9, caspase 3 and PARP 1 activity (Figure 2A). We further validated the caspase 9 activity by calorimetric assay and observed a two-fold increase of caspase 9 activity in NDRG4 knockdowned cells than the control vector and untransduced cells (Figure 2B). These results indicate induction of intrinsic like apoptosis in the absence of NDRG4 in meningioma cells.

### Targeting the NDRG4 induces mitochondrial-mediated apoptosis

**Figure 3:**
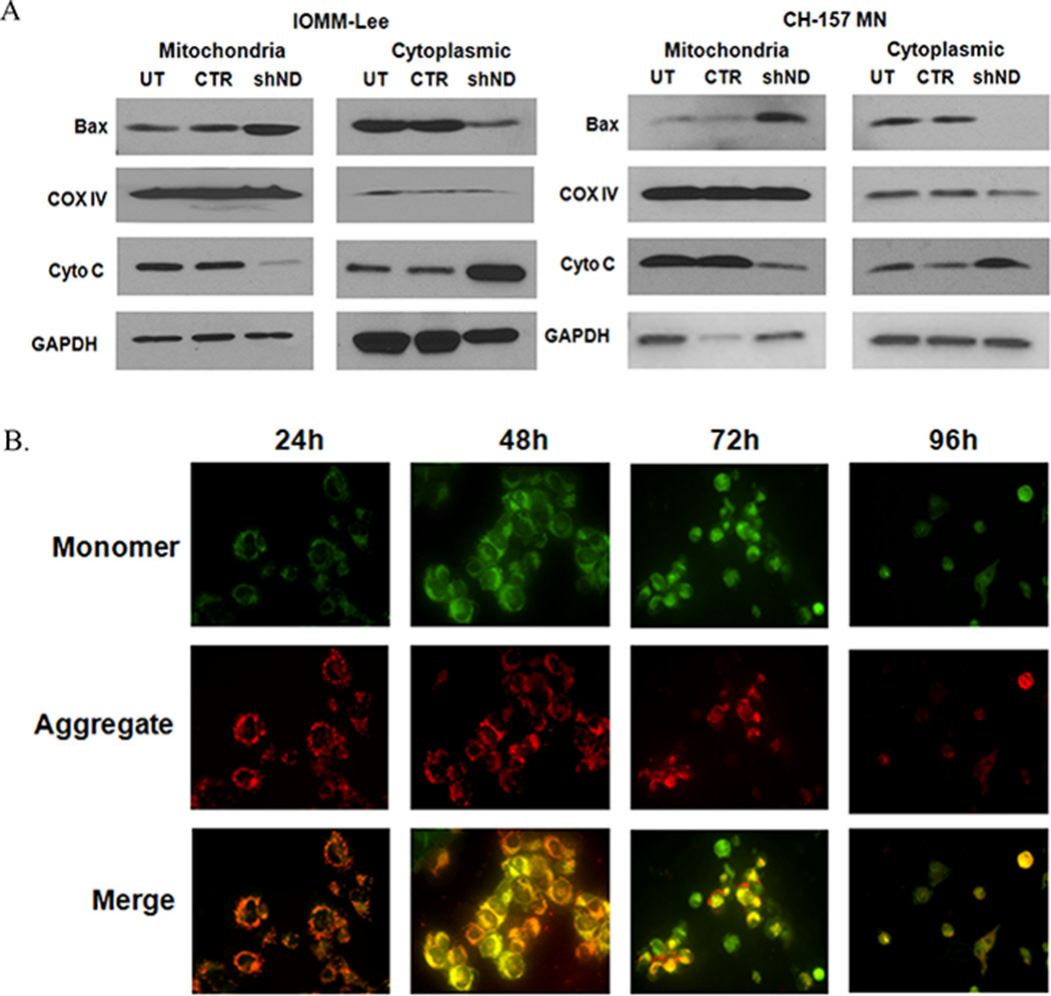
**A. Subcellular distribution of BAX and cytochrome c.** IOMM lee and CH-157 MN cells were infected with lentivirus generated in HEK293T cells transfected with control vector or NDRG4 specific shRNA. 72 hrs of post transduction, untreated, control and shNDRG4 treated cytosolic (40 μg) and mitochondrial (40 μg) protein fractions were immunoblotted-using antibodies to cytochrome c and BAX. GAPDH and COX IV were used to determine loading in cytosolic and mitochondrial fraction respectively. **B.** Mitochondrial membrane potential damage (ΔΨm). Mitochondrial membrane potential damage was evaluated using JC-1 reagent. To determine the initiation of mitochondrial apoptosis IOMM-Lee cells were infected with lentivirus generated in HEK 293T cells transfected with control vector or NDRG4 specific shRNA. After 24 hrs, 48 hrs 72 hrs and 96 hrs of post transduction, untreated, control and shNDRG4 treated cells were collected and stained with JC-1 fluorescence dye and images were captured with confocal microscopy. We carried out Immunofluorescence analysis of mitochondrial membrane potential by JC-1 staining for decreased red fluorescence (JC-1 aggregates) and increased green fluorescence (JC-1 monomers).

To determine the role of NDRG4 in mitochondrial-mediated apoptosis, we analyzed the mitochondrial and cytosolic fractions in NDRG4 knockdowned and control IOMM-Lee and CH-157 MN cells. We observed increased BAX and decreased cytochrome *c* expression in mitochondrial fractions of NDRG4 knockdowned cells when compared to control cells (Figure 3A). Correspondingly we observed that there was increased BAX and decreased cytochrome c expression in cytoplasmic fractions in control cells when compared to NDRG4 kncockdowned cells. These results suggest that BAX translocates to the mitochondria and releases cytochrome c to the cytosol leading to induction of intrinsic like apoptosis. Analyzing the lysates with COX IV and GAPDH checked the purity of the fractions. Since mitochondrial membrane potential (Ψm) damage is a key event that causes the activation of caspases, cytochrome *c* release into cytosol. We determined the Ψm using cytofluorometric lipophilic cationic dye, JC-1 by confocal microscopy. We observed more JC-1 aggregates (red fluorescence) in NDRG4 targeted cells at 24 hrs, however at later time points (72 hrs), JC-1 aggregates were diminished and JC-1 monomers (green fluorescence) increased in the cytosol (Figure 3B). At 96 hrs, both the monomeric form and dimeric forms were diminished due to apoptosis. NDRG4 targeted cells showed loss of mitochondrial membrane potential indicating mitochondrial depolarization. These results suggest that NDRG4 plays a vital role in maintaining mitochondrial membrane potential and preventing apoptosis in meningioma cells.

### NDRG4 directly regulates p53 activity

**Figure 4:**
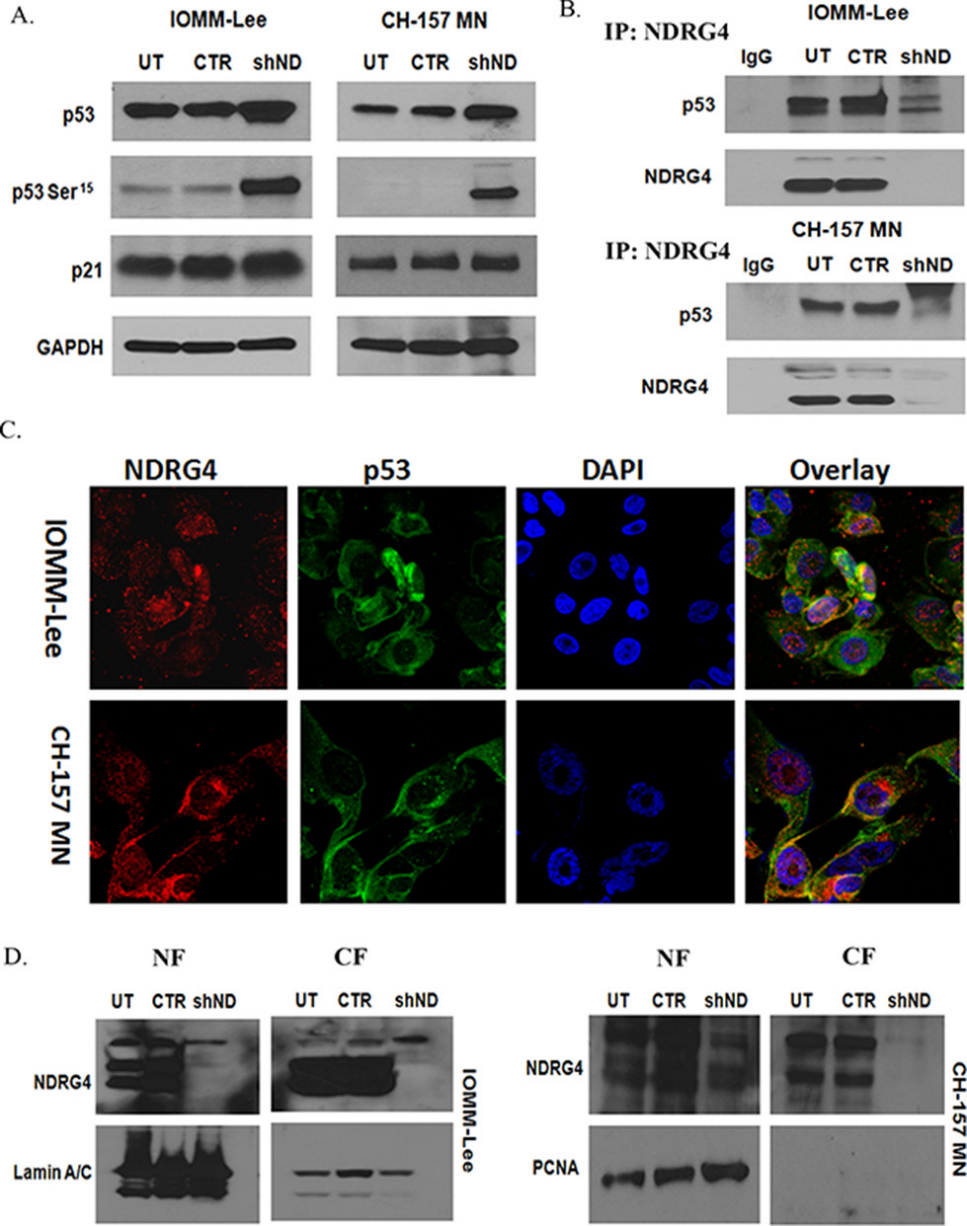
**A. Knockdown of NDRG4 enhances p53 activity.** IOMM-lee and CH-157MN cells were infected with viruses generated in HEK 293T cells transfected with Control vector or NDRG4 specific shRNA. The knockdowned cells were lysed using RIPA/MPER and 40 μg of cell lysates were immunoblotted for total p53, phospho p53 (Ser 15) and p21 protein levels. GAPDH was used to determine protein loading. **B.** NDRG4 directly interacts with p53. IOMM-lee and CH-157 MN cells were infected with lentivirus generated in HEK 293T cells transfected with control vector or NDRG4 specific shRNA. 72 hrs of post transduction, untreated, control and shNDRG4 treated total cellular protein fractions were Immunoprecipitated-using antibodies to-NDRG4 and probed with p53 antibody. **C.** P53 and NDRG4 co-localization. Immunocytochemical colocalization of NDRG4 and p53 in IOMM-Lee and CH 157-MN control cells. NDRG4 is conjugated with Alexa Fluor 594 secondary antibody. P53 is conjugated with Alexa Fluor 488 secondary antibody. Co-localization of NDRG4 and p53 was detected in IOMM-Lee and CH 157 MN control cells. All experiments were performed in triplicate (*n* = 3). **D.** Subcellular localization of NDRG4. IOMM lee and CH-157 MN cells were infected with lentivirus generated in HEK293T cells transfected with control vector or NDRG4 specific shRNA. 72 hrs of post transduction, untreated, control and shNDRG4 treated nuclear fraction (NF) (40 μg) and cytosolic fraction (CF) (40 μg) protein fractions were immunobltted-using antibodies to NDRG4. Lamin A/C and PCNA were used to determine loading in nuclear fraction.

NDRG proteins directly or indirectly associate with variety of other proteins and activate intracellular signaling pathways [13, 25, 40]. We investigated the protein levels of various pro-apoptotic proteins like p53, p21 and phospho-p53 Ser^15^ by western blotting. Targeting NDRG4 led to activation of p53 (Ser^15^) and stabilization of total p53 in both IOMM-Lee cells and CH-157 MN cells when compared to control cells (Figure 4A). Total p21 in NDRG4 targeted and control cells remained unaltered. These results indicate that p53 induces apoptosis in meningioma cells when NDRG4 is knockdowned. Since p53 was activated in NDRG4 targeted cells, we wanted to determine if there was a direct interaction between p53 and NDRG4. We performed an Immunoprecipitation assay to test for NDRG4 and p53 molecular interactions. Protein lysates of IOMM-Lee and CH-157 MN cells in the presence and absence of NDRG4 were immunoprecipated with NDRG4 antibody and probed for p53 and NDRG4 by western blotting (Figure 4B). Western immunoblotting revealed that NDRG4 indeed interacted directly with p53 and that p53 was present only in the control and untransduced lysates and absent in NDRG4 targeted lysates. Further we performed immunocytochemical staining of p53 and NDRG4 in IOMM-Lee and CH157 MN cells. Immunocytochemical analysis showed expression of both p53 (green) and NDRG4 (red) in IOMM-Lee and CH157 MN cells (Figure 4C). The overlay images revealed co-localization of p53 and NDRG4 (Figure 4C). In the fluorescence Immunocolocalization analysis of p53 and NDRG4, we observed the nuclear expression of NDRG4 in both the cell lines. To confirm the nuclear localization of NDRG4 we analyzed the NDRG4 protein levels in the nuclear and cytosolic extracts of NDRG4 knockdown and control cells by Immunoblotting (Figure 4D). Analyzing the lysates with Lamin A/C and PCNA checked the purity of the nuclear fractions. We observed NDRG4 protein expression in both the nuclear and cytosolic fractions of control cells rather than the cells knockdown of NDRG4. Comparatively with nuclear NDRG4, cytosolic NDRG4 protein expression was high in both IOMM-Lee and CH 157 MN cells. These results indicate that there is a direct interaction between NDRG4 and p53 predominantly in the cytosol.

### Inhibition of p53 attenuates NDRG4 targeted mitochondrial-mediated apoptosis

**Figure 5:**
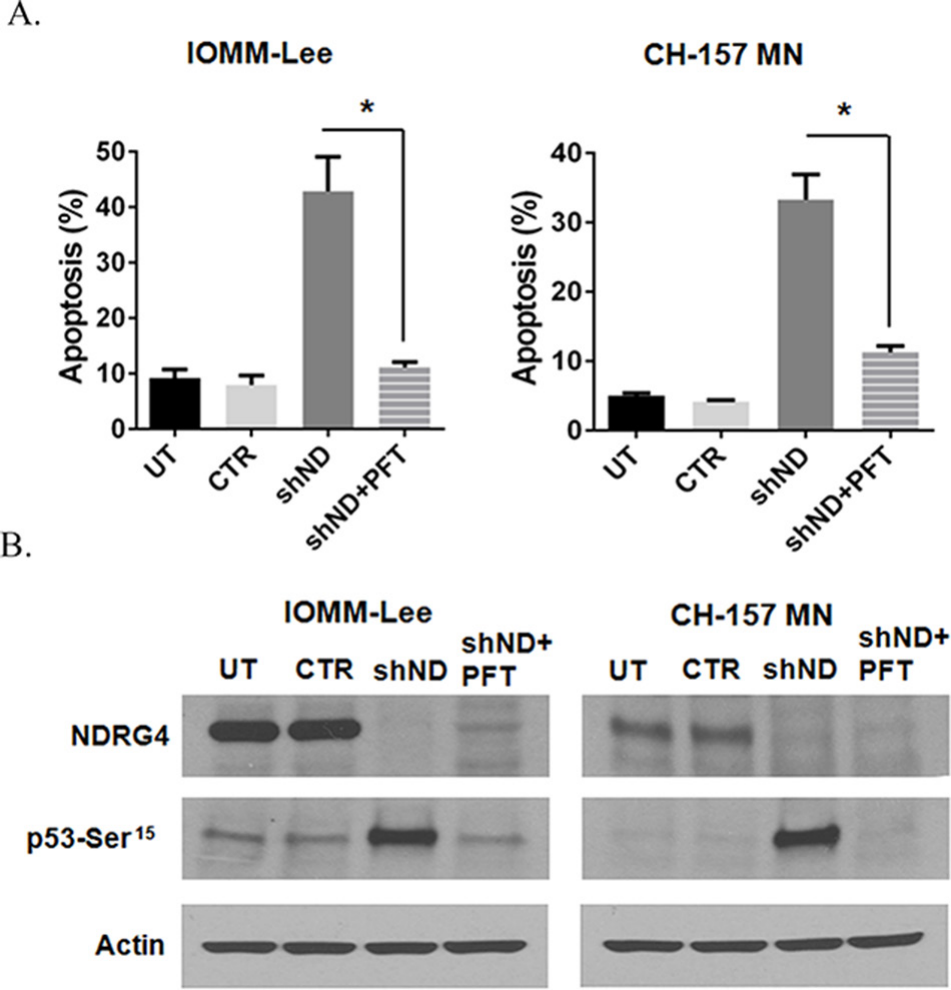
Inhibition of p53 abrogates targeted NDRG4 induced apoptosis IOMM-lee and CH-157 MN cells were infected with lentivirus generated in HEK 293T cells transfected with control vector or NDRG4 specific shRNA. PFT-α (10 μM) was added to the cells after 4 hrs of infection. 72 hrs of post transduction, untreated, control and shNDRG4 treated cells were stained with Annexin V-PE/7-AAD and analyzed by flow cytometry. **A.** The graph indicating the percentage of apoptosis for each treatment group is shown SEM of triplicates. *p < 0.05. **B.** 40 μg of the above shNDRG4 ± PFT-α treated cell lysate were immuno blotted with NDRG4 and Phospho p53 (Ser^15^). Beta actin was used to determine protein loading.

Since we observed induction and stabilization of p53 in the absence of NDRG4 and also detected direct interaction between NDRG4 and p53, we wanted to determine if p53 played a direct role in NDRG4-mediated apoptosis in meningioma cells. We inhibited p53 using PFT-α and analyzed apoptosis in meningioma cells in the presence and absence of NDRG4. Targeting NDRG4 significantly induced the apoptosis in both IOMM-Lee cells (43%; *p* = 0.0366) and CH-157 MN (33%; *p* = 0.0275) when compared to controls (Figure 5A). Inhibition of p53 by PFT-α (10 μM) abrogated the shNDRG4 induced apoptosis in IOMM-Lee (11%; *p =* < 0.05) and CH-157 MN (11%; *p* < 0.05) cells (Figure 5A). To evaluate the role of p53 in the absence of NDRG4 induced apoptosis, cells were pre-treated with Pifithrin-α (PFT-α) in the various conditions, and analyzed by Western blotting. Western immunoblots showed activation of p53 when NDRG4 was targeted in both IOMM-Lee and CH-157 MN cells (Figure 5B), and this activation was abrogated when p53 was inhibited with PFT-α (10 μM). Taken together these results indicate that inhibition of p53 prevented mitochondrial apoptosis in NDRG4 targeted meningioma cells.

## DISCUSSION

The management of recurrent and high grade meningiomas remains a challenge. The key is to elucidate disease mechanisms, validate therapeutic targets, and develop new therapeutic strategies. We believe that our studies evaluating the role of NDRG4 in meningioma biology are important for meeting that challenge.

NDRG4 plays varied roles in tumor suppression and progression in various cancers [29] [28, 41]. In our previous studies, we demonstrated that NDRG4 was overexpressed in IOMM-Lee and CH-157 MN cells and NDRG4 silencing inhibited cell proliferation, motility, invasion and angiogenesis in high grade meningioma cell lines [9]. In this study we demonstrate the molecular mechanisms of cell death associated with NDRG4 gene knockdown.

Proliferation and apoptotic cell death are complex processes involving many genes. Given the increased expression levels in aggressive meningiomas, studies were best undertaken first with NDRG4 protein depletion. We utilized lentiviral-mediated short hairpin RNA interference (52 MOI) to knockdown expression of NDRG4. In this manner, we demonstrated robust cellular death upon NDRG4 gene knockdown within the IOMM-Lee and CH-157 MN cell lines. NDRG4 gene knockdown significantly induced apoptosis as determined by FACS analysis (Figure 1B). This cell death was in the form of apoptosis as seen in GBM [29], and we proceeded to characterize the form of apoptosis.

Mitochondria represent the central checkpoint in apoptotic signaling [42, 43, 44]. Following NDRG4 silencing, we observed loss of mitochondrial membrane potential in NDRG4 targeted IOMM-Lee and CH-157 MN cell lines (Figure 3B), consistent with membrane depolarization. We then characterized the signaling associated with mitochondrial dependent apoptosis. NDRG4 gene knockdown resulted in decreased expression of Bcl-2 and BcL-xL, translocation of activated BAX into the mitochondria, cytochrome c release and enhanced cleaved caspase expression in meningioma cells (Figure 3A).

P53 overexpression is reported in several human malignant tumors [45] and elevated levels of wild type p53 are observed in aggressive malignant meningiomas [32]. P53 mediated BAX activation and induction of caspases, and apoptosis execution has been identified in multiple diseases including cancer [46, 47, 48]. Moreover, p53 has been linked to the mitochondrial intrinsic like apoptotic pathway by novel transcription-independent interactions with antiapoptotic and proapoptotic members of the Bcl-2 family proteins [49, 50]. Activated p53 can induce the expression of p21, which participates in cell cycle arrest, BAX expression, mitochondrial membrane potential damage and activation of caspases that lead to apoptosis [51]. In NDRG4 depleted meningioma we observed increased p53 activity and stabilization which activated effector caspases by releasing mitochondrial cytochrome *c* into the cytoplasm. NDRG4 gene knockdown induced apoptosis was associated with p53 phoshorylation at Ser^15^ without a change in total p53 or p21 expression levels (Figure 4A). Similar data showing apoptotic cell death was reported in embolized meningiomas [52].

NDRG4 gene knockdown results in BAX translocation in an indirect manner. According to the existing reports on the p53 intrinsic apoptotic pathway, phosphorylated p53 induces the BAX stimulation [53, 54]. Bcl_-2_ inhibits BAX dimerization and translocation into mitochondria. In our studies we have identified Bcl-2 inhibition (Figure 2) in the absence of NDRG4. These indirect downstream signaling of apoptotic response is mediated by p53. However, p53 is activated after the dissociation from NDRG4. We demonstrate that p53 activation occurs in the absence of NDRG4 (Figure 4A), that there is a direct interaction between NDRG4 and p53. These results were clearly showed in the protein-protein interaction studies (Figure 4B, C). However, there is much data demonstrating mitochondrial dependent apoptotic signaling downstream of p53 activation. In terms of BAX translocation and its mechanism [53] and downstream apoptosis signaling [54, 55].

NDRG4 gene knockdown results in intrinsic like apoptosis and our data suggest that this is p53 dependent. Similar reports of NDRG proteins roles in p53 mediated apoptosis are present in various cancers [25, 26, 56]. We demonstrated that p53 and NDRG4 co-localize, and immunoprecipitation experiments show p53 in the lysates of controls cells but not in the NDRG4 depleted lysates (Figure 4B, 4C). This suggests a possibly direct role of NDRG4 in interaction with p53 and indirect role in downstream signaling of apoptotic response. Further we have demonstrated the subcellular levels of NDRG4 (Figure 4D) because of nuclear localization of NDRG4 was observed in the fluorescence colocalization studies. These resulted confirmed the nuclear and cytoplasmic localization of NDRG4. Whereas p53 and NDRG4 interaction was much observed in the cytoplasm (Figure 4C). We believe that the cytoplasmic NDRG4 and p53 interaction was playing a crucial role in the regulation of apoptosis. In addition, we demonstrated that NDRG4 depleted meningioma cells treated with Pifitrhin-α which inhibits p53 activity, observed the significant inhibition of apoptotic cell death by Annexin-V assays. Furthermore, protein expression analysis of Phospho-p53 in PFT-α treated cells in the presence or absence of NDRG4 confirmed the role of p53 activity in triggering the apoptotic signal.

In summary, this is the first report demonstrating that NDRG4 silencing induces apoptotic cell death in aggressive meningioma cell lines. These findings advance our understanding of the role of NDRG4 in tumor progression; suggest a role for NDRG4 as a novel oncogenic protein, and as a potential therapeutic target for a difficult disease.

## MATERIALS AND METHODS

### Cells lines and chemicals

Human meningioma cancer cell lines IOMM-Lee and CH-157 MN were kindly provided by Dr. Yancie Gillespie, University of Alabama in Birmingham and Dr. Anita Lal – University of California, San Francisco and grown in DMEM, DMEM/F12 media containing 10% fetal bovine serum and 1% penicillin/streptomycin. All cell lines were maintained in a 37°C incubator in a 5% CO_2_-humidified atmosphere.

NDRG4 specific shRNA sequence (CAAACTAT GCTTCAACACCTT) and control shGFP sequence (CGAC GTAAACGGCCACAAGTT) cloned into a lentiviral packaging vector (pLKO.1) were obtained from the Genome Institute at the Washington University School of Medicine in St Louis, USA. Lentiviral packaging (pCMVdR8.2dvpr) and envelope (pCMV-VSV-G) vectors and were obtained from Adgene, Cambridge, MA. Antibodies to NDRG4 (39) V: sc-100788, p53 (D)-1): sc-126, p53 (3H2821): sc-56179), p53 (FL-393) sc-6243, p21 (F5): sc-6246, BAX(N20): sc-493, BAX(B-9): sc-7480, Bcl-xL (H-5): sc-8392, Bcl-2 (N-19): sc-492, PARP-1 (H-250): sc-7150, β-Actin (c4); sc-47778, GAPDH(A-3): sc-137179 and Pifitrhin-α were obtained from Santa Cruz Biotechnology. Anti-p53 (ab2433) was purchased from Abcam. p53(1C12) Mouse mAb #2524, Phospho-p53 (Ser^15^) (16G8) Mouse Ab #9286, Caspase-9 (mouse specific) #9504, cleaved Caspase-3 (Asp175) #9661, COX IV antibody #4844 and Anti Rabbit HRP-linked IgG #7074B were obtained from Cell Signaling. Alexafluor 488 anti mouse-Ab, was from Invitrogen. HRP Goat anti-mouse IgG antibody was from BioLegend.

### Lentiviral transduction and knockdown of NDRG 4 in meningioma cell lines

Recombinant lentiviral particles were produced in HEK293-T cells by co-transfecting lentiviral (LV) plasmid containing NDRG4 or control shRNA, along with the packaging vector pCMVdR8.2dvpr, and a plasmid encoding the vesicular stomatitis virus coat envelope pCMV-VSV-G. Lentiviral purification and infection of target cells were done as described earlier [57, 58]. Briefly IOMM-Lee and CH-157 MN cells were infected with medium containing virus (shNDRG4 or control shRNA) along with Polybrene (8 μg/ml) to enhance infectivity (16 hrs). A multiplicity of infection (MOI) of 52 was used for our experiments. Cells were washed with PBS and replenished with regular growth medium for 48–72 hrs. The transduced cells were selected by using 2 μg/ml Puromycin [9].

### Cell proliferation assay

Cell proliferation was evaluated using the 3-(4, 5-dimethylthiazol-2-yl)-2, 5-diphenyltetrazolium bromide dye method. Equal numbers (20, 000) of IOMM-Lee and CH MN 157 cells were seeded in a volume of 100 μL in each well of 96-well plate for 24 hrs. Cells were infected with viruses generated in HEK293T cells transfected with GFP or NDRG4 specific shRNA for 24 hrs, 48 hrs and 72 hrs. After incubation, MTT was added into each well to a final concentration of 5 mg/ml. The insoluble Formosan was collected and dissolved in dimethylsulfoxide (DMSO, 0.5%) and absorbance was measured at 570 nm (Bio-Tek, ).

### Immunoblot analysis

The treated cells were washed with PBS, and lysed using M-PER mammalian protein extraction reagent with protease inhibitor cocktail. The lysates were homogenized by sonication and protein concentration was determined using the BCA protein assay reagent. Cellular protein (40 μg) was separated using 10–14% SDS-PAGE and transferred onto PVDF membranes. Proteins were analyzed with specific primary antibodies and developed using a horseradish peroxidase-conjugated anti rabbit or anti-mouse IgG secondary antibody. Specific proteins were detected with western lighting Plus - ECL and using Bio-Rad Image lab images were scanned.

### Isolation of nuclear and cytosolic extracts

Nuclear and cytosolic extracts from the treated cells were isolated using Chemicon International nuclear extraction kit. Cells were washed once with 1X ice cold PBS, the cell pellet was resuspended in 1X cytosolic lysis buffer and incubated on ice for 15 min and homogenized the cells using 20–25 times with 27 gauge needle syringe. The lysates were centrifuged at 8000 g for 20 min at 4°C. The supernatant that included the cytosolic portion of the cell lysate was collected. The remaining pellet containing the nuclear portion of the cell lysates were suspended in ice cold nuclear extraction buffer and homogenized. The nuclear suspension was centrifuged at 16000 g for 5 min at 4°C to collect the nuclear fraction. Immunoblot analysis was performed with the cytoplasmic and nuclear fractions for proteins like NDRG4, Lamin A/C and PCNA.

### Isolation of mitochondrial and cytosolic extracts

Mitochondrial and cytosolic extracts from the treated cells were isolated using Active Motif mitochondrial fractionation kit. Cells were washed once with 1X ice cold PBS, the cell pellet was resuspended in 500 μl of 1X cytosolic buffer incubated for 15 min at 4^°^C on a rocking platform and homogenized the cells using 20–25 times with 0.5 ml syringe. The lysates were centrifuged at 3000 rpm for 20 min at 4°C. The supernatant that included the cytosolic and mitochondrial fraction was collected. The supernatant was centrifuged at 10,000 rpm for 20 minutes at 4°C to separate the mitochondria as a pellet and cytosolic fraction as the supernatant. The Mitochondrial pellet was washed with 100 μl 1X cytosolic buffer and centrifuged at 10,000 rpm for 10 min at 4°C to collect the mitochondrial pellet and dissolved in 100 μl of complete mitochondrial buffer. Immunoblot analysis was performed with the cytoplasmic and mitochondrial fractions for proteins like BAX, COX IV, Cytochrome C and GAPDH.

### Mitochondrial membrane potential damage

IOMM-Lee cells (2 × 10^5^) were infected with lentivirus containing shNDRG4 for 24 hrs, 48 hrs, 72 hrs and 96 hrs. Cells were then stained with 2.5 μM JC-1 (5, 5, 6, 6-tetrachloro-1, 1, 3, 3-tetraethylbenzimidazolylcarbocyanineiodide, Cayman Chemicals) for 30 min at 37°C, and Digital pictures for JC monomers (Green fluorescence; 535 nm) and JC aggregates (red fluorescence; 570 nm ) were captured by confocal microscope (Zeise LSM 510).

### Immunoprecipitation

Cell lysates (400 μg) from shGFP and shNDRG4 transduced cell lines were incubated with NDRG4 specific antibody (2 μg) overnight at 4°C. Following incubation 30 μl of protein G coupled magnetic beads (MACS) were added to antibody conjugated protein and incubated for 30 min on ice. The immunoprecipitated protein lysate complex was then added to the pre-equilibrated micro columns (miltenybiotec) and washed with lysis buffer and eluted with 1X SDS sample buffer containing β-Mercaptoethanol at 90°C. The Immunoprecipitated protein was resolved on SDS-PAGE and immunoblotted for p53 and NDRG4.

### Immunocytochemical co-localization analysis

IOMM-Lee and CH-157 MN cells (5000) seeded on 4-well chamber slides were fixed with 4% paraformaldehyde and incubated with 1% bovine serum albumin in PBS at room temperature for 30 min. Cells were washed with PBS, and incubated overnight with primary antibodies (1:100), at 4°C. Cells were then washed with PBS, and incubated with secondary antibodies for 1 hr at room temperature. The cells were then washed, and mounted with an anti-fade reagent containing DAPI and fluorescent photomicrographs were then obtained by confocal microscope (Zeiss LSM 510).

### Caspase-9 activity assay

A quantitative Caspase 9 enzyme activity assay was performed using Caspase 9 assay kit (Bio-vision). Cell lysates (200 μg) were incubated with 50 μl of 2X reaction buffer and 5 μl of the 4 mM LEHD-*p*NA substrate and incubated at 37°C for 1–2 hrs. The caspase activity was measured as the *p*NA light emission at 405-nm.

### Apoptosis assay

Apoptosis was determined using apoptosis detection kit (BD Pharmingen, USA). Cells (100,000) were incubated with Annexin V-APC/PE and propidium iodide/7-AAD for 15 min at room temperature. The cells were then analyzed by flow cytometry, using a two-color FACS analysis system (BD LSR II). For each treatment, the average fold-increase of apoptotic cells over control was calculated.

### Statistical methods

Statistical analysis and graphical presentation was done using quantitative data from MTT assay, FACS analysis and other assays were evaluated for statistical significance using GraphPad Prism 4.0. Data for each treatment group were represented as means ± SEM and compared with other groups for significance by one-way ANOVA. Results presented in this study are the representative images of three independent experiments (*n* = 3) and results are expressed as mean ± SEM. and differences were considered significant at a *p* value of less than 0.05.
